# Collagen molecular phenotypic switch between non-neoplastic and neoplastic canine mammary tissues

**DOI:** 10.1038/s41598-021-87380-y

**Published:** 2021-04-21

**Authors:** Masahiko Terajima, Yuki Taga, Becky K. Brisson, Amy C. Durham, Kotaro Sato, Katsuhiro Uzawa, Tomoaki Saito, Shunji Hattori, Karin U. Sørenmo, Mitsuo Yamauchi, Susan W. Volk

**Affiliations:** 1 Division of Oral and Craniofacial Health Sciences, Adams School of Dentistry, University of North Carolina at Chapel Hill, Chapel Hill, USA; 2 Nippi Research Institute of Biomatrix, Tokyo, Japan; 3 Department of Clinical Sciences and Advanced Medicine, School of Veterinary Medicine, University of Pennsylvania, Philadelphia, USA; 4 Department of Pathobiology, School of Veterinary Medicine, University of Pennsylvania, Philadelphia, USA; 5 Department of Oral and Maxillofacial Surgery, Nagoya University Graduate School of Medicine, Nagoya, Japan; 6 Department of Oral Science, Graduate School of Medicine, Chiba University, Chiba, Japan; 7 Department of Biomedical Sciences, School of Veterinary Medicine, University of Pennsylvania, Philadelphia, USA

**Keywords:** Breast cancer, Cancer microenvironment, Cancer models, Cancer therapy, Mechanisms of disease, Post-translational modifications

## Abstract

In spite of major advances over the past several decades in diagnosis and treatment, breast cancer remains a global cause of morbidity and premature death for both human and veterinary patients. Due to multiple shared clinicopathological features, dogs provide an excellent model of human breast cancer, thus, a comparative oncology approach may advance our understanding of breast cancer biology and improve patient outcomes. Despite an increasing awareness of the critical role of fibrillar collagens in breast cancer biology, tumor-permissive collagen features are still ill-defined. Here, we characterize the molecular and morphological phenotypes of type I collagen in canine mammary gland tumors. Canine mammary carcinoma samples contained longer collagen fibers as well as a greater population of wider fibers compared to non-neoplastic and adenoma samples. Furthermore, the total number of collagen cross-links enriched in the stable hydroxylysine-aldehyde derived cross-links was significantly increased in neoplastic mammary gland samples compared to non-neoplastic mammary gland tissue. The mass spectrometric analyses of type I collagen revealed that in malignant mammary tumor samples, lysine residues, in particular those in the telopeptides, were markedly over-hydroxylated in comparison to non-neoplastic mammary tissue. The extent of glycosylation of hydroxylysine residues was comparable among the groups. Consistent with these data, expression levels of genes encoding lysyl hydroxylase 2 (LH2) and its molecular chaperone FK506-binding protein 65 were both significantly increased in neoplastic samples. These alterations likely lead to an increase in the LH2-mediated stable collagen cross-links in mammary carcinoma that may promote tumor cell metastasis in these patients.

## Introduction

Fibrillar collagens are the most abundant component within the extracellular matrix (ECM) of the tumor stroma and have been increasingly recognized for their ability to modulate the biologic behavior of breast cancer. Notably, the ability of tumor cells to sense the biophysical and biomechanical features of collagen is recognized as a key mechanism which promotes events associated with metastasis and chemoresistance^1,2^. Breast cancer is the most frequently diagnosed cancer in both women and sexually intact female dogs, a devastating cancer for both women and veterinary patients^3–8^. Spontaneously occurring canine mammary gland tumors (CMT) have been proposed to have advantages as a model of human breast cancer compared to murine models, particularly when examining the tumor stroma^9–11^. In fact, recent work supports similarities between prognostic collagen signatures of women and female dogs and cats^12–16^, indicating that a comparative oncology approach provides valuable insights into the role of collagen in directing clinical behavior of breast cancer of veterinary and human patients. Recent studies have shown that aberrant collagen cross-linking is in part responsible for stiffening tumor ECM that facilitates cancer progression and metastasis^1,17,18^. Much attention has been paid to a significant role of several collagen modifying enzymes that determine the quantity and quality of collagen cross-linking in these processes; however, their roles in spontaneous large animal models of breast cancer have yet to be defined.

Type I collagen is a heterotrimeric molecule composed of two α1 chains and one α2 chain, and the molecule consists of three structural domains: amino-terminal nonhelical telopeptide (N-telo), central triple helical (helical), and carboxy-terminal nonhelical telopeptide (C-telo) domains^19^. A functionally important feature of this protein is a series of unique post-translational modifications that occur in- and outside of the cell. The intracellular modifications include hydroxylation of specific lysine (Lys) and proline (Pro)^20^ residues, and *O*-linked mono- and di-glycosylation of specific hydroxylysine (Hyl) residues. In the extracellular space, upon removal of the N- and C-propeptide extensions, collagen molecules are packed into a fibril and stabilized by covalent intermolecular cross-links (see below)^19^. A number of collagen-specific enzymes and associated chaperones are involved in these highly complicated, multi-step processes of post-translational modifications^21^.

In the endoplasmic reticulum (ER), Lys hydroxylation of procollagen α chains is catalyzed by lysyl hydroxylases 1–3 (LH1-3). It occurs in the X-Lys-Gly sequence within the triple helical domain, and in the X-Lys-Ser/Ala sequence in the N- and C-telo domains of the procollagen α chains, respectively. LH1 is the main helical LH while LH2 is the specific telopeptidyl LH^22–24^. Glycosylation of Hyl is catalyzed by glycosyltransferase 25 domain 1 and 2 (GLT25D1, 2) to form galactosyl-Hyl (G-Hyl) and then by LH3 to add glucose to G-Hyl to produce glucosylgalactosyl-Hyl (GG-Hyl)^25,26^. Collagen cross-linking is initiated in the extracellular space by the oxidative deamination of Lys and Hyl residues in the N- and C-telo domains (α1-9^N^/16^C^ and α2-5^N^ in the case of type I collagen) to the respective aldehydes, Lys^ald^ or Hyl^ald^, through the action of lysyl oxidases (LOX, LOXL1-4). Once these aldehydes are formed, they initiate a series of condensation reactions involving the juxtaposed Lys, Hyl, and histidine residues. The Hyl^ald^ that is generated by modifications via LH2 and LOX/LOXLs leads to the formation of more stable cross-links in comparison to those of the Lys^ald^-derived pathway^27^. Thus, the quantity and quality/stability of collagen cross-links are mainly determined by LOX/LOXLs and LHs (LH2 in particular) activities, respectively.

It has been shown that genes encoding LOX and LOXL2 are up-regulated in various cancer types^28–30^, indicating increases in collagen cross-links in tumor samples. More recent reports have shown that not only these genes but also that encoding LH2, i.e. procollagen-lysine,2-oxoglutarate 5-dioxygenase 2 (PLOD2), is increased in breast^31,32^, sarcoma^33^, lung^34^, and glioma^35^ cancer models, indicating that the type of cross-linking is switched to a stable Hyl^ald^-derived pathway. However, collagen cross-link analyses have been performed only on limited cancer types, i.e. lung^34^ and oral cancers^18^, and these results support the early speculations. To the best of our knowledge, in any type of tumor, no studies have been done on Lys post-translational modifications at specific molecular sites of collagen. This specific information, together with the degree and type of cross-linking, are critically important as they provide insight into the molecular basis of abnormal tumor microenvironments that regulate cancer cell behavior and may inform novel, directed therapies.

Efficient translation of therapies is highly dependent on models bearing robust fidelity. Although murine models play a key role in breast cancer research, the dog has additional advantages as a model for human breast cancer, including its spontaneous development in the context of an intact immune system, and its significant similarities with human breast cancer with respect to clinical presentation, genetics, molecular marker expression, hormone dependency and disease progression^11,36–43^. In an effort to identify tumor-permissive collagen features characteristic of mammary neoplasia and specifically malignancy in this model, we examined fibrillar collagen architecture and performed in-depth analysis of the molecular and morphological phenotypes of type I collagen in canine non-neoplastic tissue, as well as mammary adenoma and carcinoma. As such, this work further validates this model with respect to the stromal microenvironment and provides critical insight that defines mechanisms driving the formation of tumor-permissive collagen signatures. Furthermore, use of this model will facilitate the assessment of the efficacy and safety of therapeutic targets that disrupt or reverse the formation of tumor-permissive collagen signatures in future studies. Notably, this “One Health” approach may identify novel therapeutic targets and provide a gateway that may more accurately predict their success through clinical trials in dogs, which ultimately would improve clinical outcome in both canine mammary tumor patients and women with breast cancer.

## Methods

### Ethics statement

This research was performed in accordance with a protocol approved by the University of Pennsylvania Institutional Animal Care and Use Committee (protocol # 804298 to KUS). Samples were obtained from excess tissues that were collected while dogs were undergoing standard of care surgical removal of mammary gland tumors or at necropsy (one patient did not have surgery and was euthanized in accordance with the current guidelines of the American Veterinary Medical Association). Informed consent was obtained from the authorized welfare advocate of each participating dog to receive standard of care veterinary diagnostics and treatment and use of excess tissues for research purposes.

### Canine cohort and mammary tissue samples

Non-neoplastic and neoplastic mammary tissue samples were collected from dogs participating in a prospective study by the Penn Vet Shelter Canine Mammary Tumor Program (PVSCMTP) at the University of Pennsylvania School of Veterinary Medicine. PVSCMTP provides standard of care staging and surgical treatments to dogs with mammary tumors and in doing so enables their placement in permanent homes. Given the lack of previous medical history of this particular patient population, exact age of patients is unknown. The breeds of 33 dogs that contributed tissues to this study, and the histopathologic diagnoses of these tissues, can be found in Supplementary Table S1. Non-neoplastic samples included both tissues without notable pathology, as well as those with evidence of lobular hyperplasia, a physiologic change in older, sexually-intact female dogs. Biopsied tissue was divided into samples for routine histopathologic interpretation of formalin-fixed paraffin embedded tissues, immediately frozen for biochemical analysis, or frozen in RNA later for expression analysis (individual patient/sample contributions for these analyses are detailed in Supplementary Table S1). For histopathology, samples were fixed in 10% formalin, paraffin embedded, processed, and serial 4-µm sections were used for second harmonic generation (SHG) imaging or stained with hematoxylin and eosin (H&E) as previously described^44^. To avoid inter-observer variability in histological interpretation that has been documented in recent studies^45,46^, all biopsies were reviewed by a single board-certified veterinary pathologist (ACD) and malignant samples categorized by grade according to the modified Elston-Ellis grading system for dogs^47^.

### SHG imaging and analysis

SHG images were taken and analyzed as previous described^12,16^. Briefly, a Leica SP8 confocal/multiphoton microscope (Leica Microsystems, Inc., Mannheim, Germany), was used to collect SHG (backward) signal. Pathologist-identified non-neoplastic, adenoma, or carcinoma regions were marked within tissue samples on serial H&E stained slides to guide SHG imaging in the required regions of unstained slides. Five SHG images (one z-plane) were taken per sample. The autofluorescence was subtracted from the original SHG images as previously described^12^ before collagen analysis. Collagen density was analyzed using Fiji Image Analysis software which measured the integrated density of each SHG image, which incorporates intensity of each pixel and % area of positive SHG signal. To analyze the collagen fiber characteristics, CT-FIRE identified collagen fibers and analyzed each fiber for length, width, and straightness. For fiber width distribution, a histogram was generated for every image, and bins were averaged per sample.

### Collagen preparation for biochemical analysis

Samples were obtained from canine mammary tissues, immediately frozen, and stored at − 80 °C for collagen biochemical analysis. The dissected samples were pulverized with a pestle and mortar to a fine powder under liquid nitrogen. Pulverized samples were washed several times with cold phosphate-buffered saline (PBS), and cold distilled water by repeated centrifugation at 4000×g for 30 min, and lyophilized.

### Site-specific characterization of post-translational modifications at telopeptidyl and triple helical modification sites of type I collagen

Collagen was extracted from the lyophilized mammary tissues by pepsin (5 mg/ml in 0.5 M acetic acid; Sigma-Aldrich, St. Louis, MO, USA) at 4 °C for 24 h and subsequently purified by salt precipitation (2 M NaCl). As previously reported^48^ the collagen samples were digested with trypsin (1:100 enzyme/substrate ratio) in 100 mM Tris–HCl, 1 mM CaCl_2_ (pH 7.6) at 37 °C for 16 h to analyze the Lys and Pro post-translational modifications at the specific molecular sites within the triple helical domain of type I collagen. In addition, to analyze Lys hydroxylation at the telopeptide domains of type I collagen, the lyophilized tissue samples were sequentially digested with bacterial collagenase and pepsin as previously reported^48^. In brief, the samples were digested with 0.01 mg/ml of recombinant collagenase from *Grimontia hollisae* (Nippi, Tokyo, Japan)^49^ in 100 mM Tris–HCl/5 mM CaCl_2_ (pH 7.5) at 37 °C for 16 h after heating at 60 °C for 30 min. After addition of acetic acid (final 0.5 M), the collagenase digests were further digested with 0.01 mg/ml of pepsin at 37 °C for 16 h. The peptide solutions digested with trypsin or collagenase/pepsin were subjected to LC-quadrupole time-of-flight (QTOF)-MS analysis on a maXis II QTOF mass spectrometer (Bruker Daltonics, Bremen, Germany) coupled to a Shimadzu Prominence UFLC-XR system (Shimadzu, Kyoto, Japan) using an Ascentis Express C18 HPLC column (5 µm particle size, L × I.D. 150 mm × 2.1 mm; Supelco, Bellefonte, PA, USA)^48^. Site occupancy of Lys hydroxylation/glycosylation (Lys, Hyl, G-Hyl, and GG-Hyl) and Pro 3-hydroxylation (Pro and 3-hydroxyproline (3-Hyp)) were calculated using the peak area ratio of extracted ion chromatograms (mass precision range =  ± 0.05 for the tryptic digests and ± 0.02 for the collagenase/pepsin digests) of peptides containing the respective molecular species as previously reported^48,50–52^ with adaptation for dog sequences.

### Reduction of collagen with NaB^3^H_4_

Dried canine mammary tissue samples (~ 2.0 mg each) were suspended in buffer containing 0.15 M N-trismethyl-2-aminoethanesulfonic acid, and 0.05 M Tris–HCl, pH 7.4, and reduced with standardized NaB^3^H_4_. The specific activity of the NaB^3^H_4_ was determined by the method described previously^53,54^. The reduced samples were washed with cold distilled water several times by repeated centrifugation at 4000×g and lyophilized.

### Amino acid and cross-link analyses

Reduced collagen was hydrolyzed with 6 N HCl and subjected to amino acid analysis as described previously^55^. The extent of Lys hydroxylation in collagen was calculated based on the value of 300 residues of Hyp per collagen molecule.

Aliquots of the hydrolysates were also subjected to cross-link analysis as described previously^55–57^. Upon reduction, the immature reducible cross-links, dehydrodihydroxylysinonorleucine (dehydro-DHLNL)/its ketoamine, dehydrohydroxylysinonorleucine (dehydro-HLNL)/its ketoamine, and dehydrohistidinohydroxymerodesmosine (dehydro-HHMD) were reduced to stable secondary amines, DHLNL, HLNL, and HHMD. Hereafter, the terms DHLNL, HLNL, and HHMD will be used for both the unreduced and reduced forms. Mature non-reducible cross-links, pyridinoline (Pyr) and deoxypyridinoline (d-Pyr), were simultaneously analyzed by their fluorescence. The levels of immature reducible (DHLNL, HLNL, and HHMD) and mature non-reducible cross-links (Pyr and d-Pyr) were quantified as mole/mole of collagen. A total number of aldehydes were calculated as a sum of DHLNL + HLNL + 2 × Pyr + 2 × d-Pyr + 2 × HHMD based on the number of aldehydes involved in cross-links^48^. The ratio of Hyl^ald^- to Lys^ald^-derived cross-links (i.e., LH2-modified to LH2-nonmodified cross-links) was calculated as (DHLNL + Pyr + d-Pyr)/HHMD^34^. The HLNL cross-link was excluded in the ratio because this can be derived from either Hyl^ald^ or Lys^ald^^19^.

### Quantitative real-time PCR

To determine the expression of Lys moxdifying enzymes and the recently proposed LH-associated molecular chaperones, quantitative real-time PCR was performed as previously described^58^. Briefly, RNA was extracted from frozen or RNA*later*-stabalized non-neoplastic and neoplastic mammary gland tissues, cDNA generated, and mRNA expression (normalized to GAPDH as the endogenous control) was determined for the following proteins: LOX, LH1-3, FK506-binding protein 65 (Fkbp65), immunoglubulin heavy-chain-binding protein (Bip), Synaptonemal Complex 65 (Sc65), and prolyl 3-hydroxylase 3 (P3H3) (see Supplementary Table S2 for primer sequences). Fold change was calculated as compared to non-neoplastic samples.

### Statistical analysis

Statistical analyses were performed using GraphPad Prism 7 (La Jolla, CA, USA). Collagen fiber characteristics or biochemical statistical differences between three groups (non-neoplastic, adenoma, and carcinoma) were determined by Kruskal–Wallis one-way analysis of variance followed by Dunn's multiple comparison tests and means between two groups were compared by Student’s *t* tests. For distribution comparisons, nonlinear regression was used to find the best-fit model and values from the resulting curves (amplitude, spread, mean) were compared via 1-way ANOVA followed by Tukey post-hoc test. Data are presented as means ± SD or ± SEM, as defined in figure and table legends, and *p* values < 0.05 were considered significant. Outliers in cross-linking and collagen fiber CT-FIRE data were identified by GraphPad Prism and removed from analysis. Sample size was determined by power analysis after preliminary results were obtained, but was limited by sample availability. This study was carried out in compliance with the ARRIVE guidelines.

## Results

### Fibrillar collagen architecture characterization in histologic samples of non-neoplastic and neoplastic canine mammary tissue

**Figure 1:**
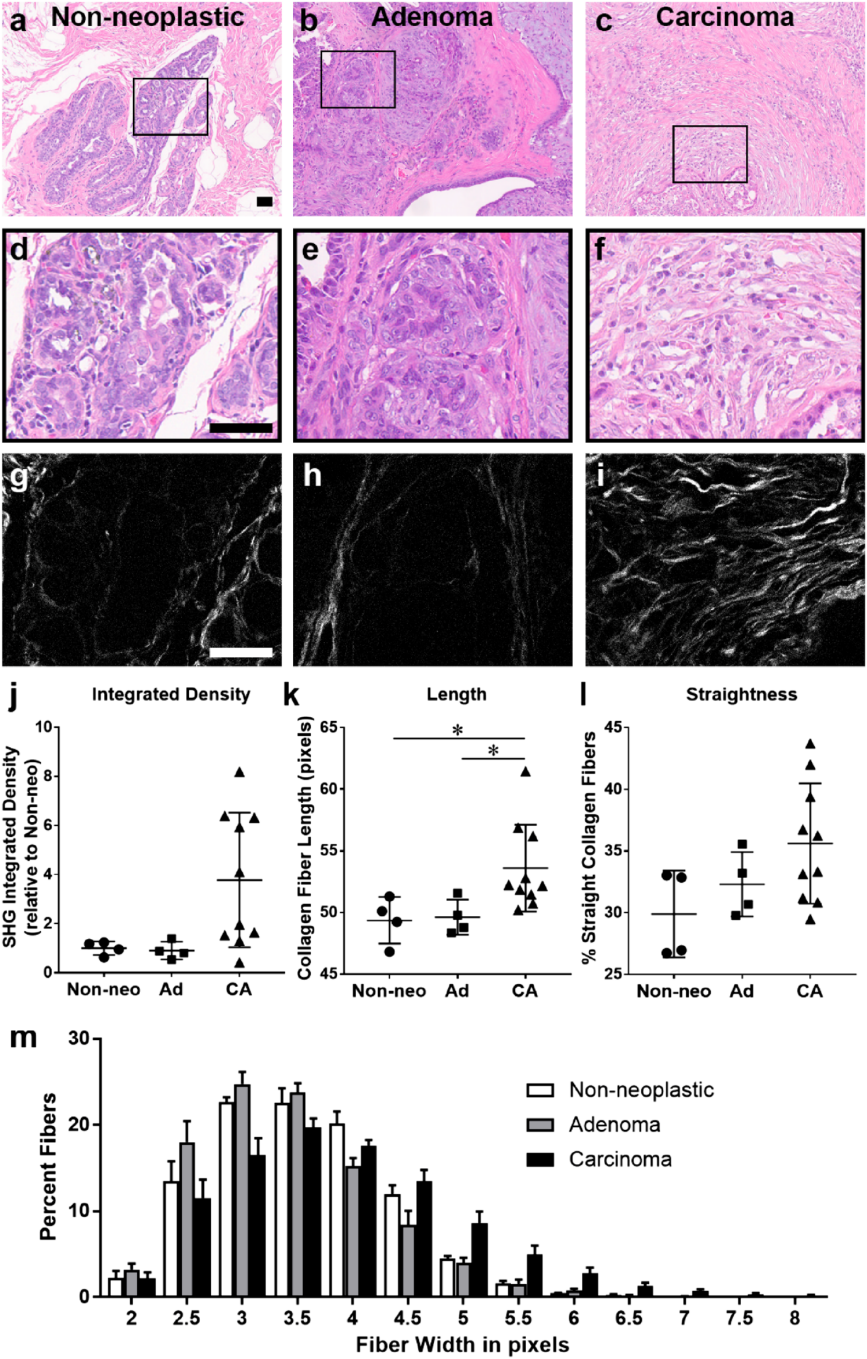
Collagen fiber characteristics in canine mammary tissue. (**a**–**f**) H&E and (**g**–**i**) second harmonic generation (SHG; collagen in white) images from non-neoplastic (**a**,**d**,**g**), adenoma (**b**,**e**,**h**) and carcinoma (**c**,**f**,**i**) samples. Scale bar = 50 µm. Black boxes in **a**–**c** correspond to higher magnification images in **d**–**f**. (**j**–**l**) Fibrillar collagen signal (integrated density; **j**) as well as fiber length (**k**), straightness (**l**) and width (**m**) was analyzed using the CT-FIRE program (Non-neo: Non-neoplastic n = 4; Ad: Adenoma n = 4; CA: Carcinoma n = 10). Data are presented as means ± SD. The three tissue types were compared using Kruskal–Wallis followed by Dunn’s multiple comparison tests (*, *p* < 0.05). (**m**) To compare fiber width distributions, histograms were generated (presented as means ± SEM). Non-linear regression was used to find the best fit model for each tissue type and curve values (amplitude, spread, mean) were compared using 1-way ANOVA followed by a Tukey post-hoc test (non-neoplastic vs carcinoma: curve amplitude *p* = 0.004, curve spread *p* = 0.011, curve mean *p* = 0.007; adenoma vs carcinoma: curve amplitude *p* < 0.001, curve spread *p* = 0.001, curve mean *p* < 0.0001).

The architectural features of the fibrillar collagen matrix, including density and collagen fiber characteristics (width, length, straightness), were examined by label-free two photon SHG imaging. Areas of non-neoplastic mammary tissue, mammary adenoma and mammary carcinoma were identified from H&E-stained histologic slides obtained from biopsy samples from dogs undergoing treatment for mammary tumors and fibrillar collagen was imaged in corresponding regions using SHG microscopy (representative images, Fig. 1a–i). In mammary tissue, both non-neoplastic and adenoma samples contained collagen fibers that were thin and surrounded the mammary glands, although there was expansion of neoplastic epithelial cells within surrounding areas of collagen in adenoma samples compared to normal glandular tissue. In contrast, carcinoma samples had disruption of the regular collagen-tumor architecture by infiltrating neoplastic cells and an increase in stroma volume, leading to a lack of a discrete tumor stromal boundary; however, there was significant intra and inter-tumoral heterogeneity with respect to amount of collagen present. When the individual fiber characteristics were analyzed in SHG images using the CT-FIRE program, significant differences were noted associated with malignancy, while values between non-neoplastic and adenoma samples were similar (Fig. 1j–m). Although there was a trend for increases in both overall fibrillar collagen signal (integrated density) and fiber straightness in carcinoma samples compared to non-neoplastic samples, these differences were not significant. Fiber length was significantly increased in carcinoma samples compared to both non-neoplastic and adenoma mammary samples (Fig. 1k; *p* < 0.034 and *p* < 0.042, respectively). While several collagen fiber characteristics predicted survival time in dogs with mammary carcinoma, the robust ability of increased collagen fiber width to independently predict poor survival in a multivariate model^12^ prompted a detailed examination of the populations of fiber widths found within the three groups. The fibers in non-neoplastic and adenoma samples had similar patterns of collagen fiber width distribution. In contrast, carcinoma samples contained a more diverse population including a larger proportion of wider fibers (represented by a flattened and right-shifted distribution in Fig. 1m; non-neoplastic vs carcinoma: curve amplitude *p* = 0.004, curve spread *p* = 0.011, curve mean *p* = 0.007; adenoma vs carcinoma: curve amplitude *p* < 0.001, curve spread *p* = 0.001, curve mean *p* < 0.0001). This data indicates a broad distribution of collagen fiber widths with significantly greater numbers of wide fibers present in carcinoma samples that were not in non-neoplastic or adenoma samples.

### Lysine hydroxylation

To further characterize the collagen matrix of non-neoplastic and neoplastic mammary tissue at the biochemical level, the extent of Lys hydroxylation of collagen was analyzed as previously reported^26^. In both mammary carcinoma and adenoma samples, Lys hydroxylation were significantly increased by ~ 32–39% compared with that of non-neoplastic mammary tissue (Table 1, *p* < 0.01). Neoplastic samples (adenoma and carcinoma combined) had an overall 34.7% increase in Lys hydroxylation relative to non-neoplastic samples (Table 1, *p* < 0.001). These results indicate that, in canine neoplastic mammary tissue, Lys residues in a collagen molecule including the helical and telopeptide domains^19^ are over-hydroxylated compared to non-neoplastic mammary tissue.

**Table 1:** Hydroxylation of lysine (Lys) in collagen from canine mammary tissues.

Non-neoplastic	Adenoma	Carcinoma	Neoplastic
11.8 ± 0.7	16.4 ± 2.2**	15.6 ± 2.0**	15.9 ± 2.1^###^

Values represent mean hydroxylated residues/mole of collagen ± SD (non-neoplastic, n = 5; adenoma, n = 7; carcinoma, n = 9; neoplastic, n = 16).

**p < 0.01 between non-neoplastic and benign (adenoma) or malignant (carcinoma) mammary gland tumors via Kruskal–Wallis followed by a Dunn’s multiple comparisons test.

^###^*p* < 0.001 between non-neoplastic and neoplastic (combined adenoma and carcinoma) mammary gland tumors via an unpaired Student’s t-test.

### Alterations of collagen post-translational modifications at specific molecular loci of the helical domain of type I collagen

Post-translational modifications including Pro 3-hydroxylation (P3H), Lys hydroxylation, Hyl glycosylation (G-Hyl, GG-Hyl) at specific sites of type I collagen were analyzed by LC-QTOF-MS (Table 2) using tryptic digests of collagen purified from non-neoplastic and neoplastic mammary tissue samples, as previously reported^48,50–52^. The extents of P3H were essentially identical in non-neoplastic and neoplastic samples with no statistical differences at the major modification sites, i.e. α1(I) Pro-986/707 and α2(I) Pro-707 (*p* > 0.05).

**Table 2:** Summary of site-specific modification analysis by mass spectrometry of non-cross-linked, hydroxylated and glycosylated residues in the triple helical domain of type I collagen from canine non-neoplastic and neoplastic mammary tissue samples.

	Site occupancy (%)	
	Non-neoplastic	Neoplastic
**α1(I) Pro-986**		
Pro	2.30 ± 0.8	2.8 ± 1.3
3-Hyp	97.7 ± 0.8	97.2 ± 1.3
**α1(I) Pro-707**		
Pro	93.2 ± 0.8	92.3 ± 4.0
3-Hyp	6.8 ± 0.8	7.7 ± 4.0
**α2(I) Pro-707**		
Pro	89.3 ± 1.2	88.6 ± 6.8
3-Hyp	10.7 ± 1.2	11.4 ± 6.8
**α1(I) Lys-87**		
Lys	0.0 ± 0.0	0.0 ± 0.0
Hyl	0.0 ± 0.0	0.0 ± 0.0
G-Hyl	18.3 ± 1.9	22.6 ± 5.2
GG-Hyl	81.7 ± 1.9	77.4 ± 5.2
**α1(I) Lys-99**		
Lys	76.9 ± 2.5	75.8 ± 6.2
Hyl	19.4 ± 2.2	19.7 ± 5.2
G-Hyl	2.7 ± 0.2	3.3 ± 0.9
GG-Hyl	1.1 ± 0.3	1.2 ± 0.8
**α1(I) Lys-174**		
Lys	49.3 ± 2.9	34.6 ± 8.5^#^
Hyl	44.2 ± 2.2	57.2 ± 6.6^##^
G-Hyl	5.5 ± 0.4	6.8 ± 1.7
GG-Hyl	1.0 ± 0.2	1.4 ± 0.9
**α1(I) Lys-219**		
Lys	84.3 ± 2.3	79.8 ± 4.4
Hyl	15.7 ± 2.3	20.3 ± 4.4
**α1(I) Lys-564**		
Lys	85.6 ± 1.0	74.1 ± 5.6^##^
Hyl	12.6 ± 0.7	23.4 ± 5.0^##^
G-Hyl	1.2 ± 0.3	2.0 ± 0.6
GG-Hyl	0.6 ± 0.1	0.5 ± 0.3
**α2(I) Lys-87**		
Lys	2.5 ± 0.7	1.4 ± 0.6^#^
Hyl	0.9 ± 0.4	0.8 ± 0.2^#^
G-Hyl	11.2 ± 2.1	16.2 ± 3.2^#^
GG-Hyl	85.4 ± 2.9	81.6 ± 3.4
**α2(I) Lys-174**		
Lys	13.3 ± 1.1	7.7 ± 3.4^#^
Hyl	2.2 ± 0.6	1.8 ± 1.3
G-Hyl	72.5 ± 1.8	80.9 ± 3.9^##^
GG-Hyl	11.9 ± 1.9	9.7 ± 2.6
**α2(I) Lys-219**		
Lys	19.5 ± 1.3	12.8 ± 3.7^#^
Hyl	46.6 ± 2.1	57.9 ± 11.5
G-Hyl	12.3 ± 0.2	12.4 ± 4.0
GG-Hyl	21.6 ± 3.2	16.9 ± 6.7
**α1(I) Lys-918/930**		
Lys + Lys	0.9 ± 0.1	0.8 ± 0.4
Lys + Hyl	8.5 ± 0.4	6.5 ± 1.6
Hyl + Hyl	90.6 ± 0.4	92.8 ± 1.9

Lys hydroxylation and its glycosylation (%) represents the relative levels of Lys, Hyl, G-Hyl, and GG-Hyl (Lys + Hyl + G-Hyl + GG-Hyl = 100%), and Pro 3-hydroxylation represents the relative levels of Pro and 3-Hyp (Pro + 3-Hyp = 100%). Pro, proline; 3-Hyp, 3-hydroxyproline; Lys, lysine; Hyl, hydroxylysine; G-, galactosyl-; GG-, glucosylgalactosyl.

Values represent mean percentages ± S.D. (non-neoplastic, n = 3; neoplastic, n = 9).

^#^*p* < 0.05, ^##^*p* < 0.01 between non-neoplastic and neoplastic via unpaired Student’s t-tests.

However, Lys modifications in the triple helical region of type I collagen were significantly altered in the neoplastic mammary tumor samples in a site-specific manner compared to non-neoplastic samples (Table 2). Lys hydroxylation of the α1 chain was significantly increased in neoplastic samples compared to non-neoplastic tissues at α1 Lys-174 (44.2% for non-neoplastic and 57.2% for neoplastic; *p* < 0.01) and α1 Lys-564 (12.6% for non-neoplastic and 23.4% for neoplastic; *p* < 0.01). Lys was completely hydroxylated and glycosylated at α1 Lys-87 in all non-neoplastic and neoplastic mammary tissue samples (Lys = 0% and Hyl = 0%). For α1 Lys-930, one of the helical cross-linking sites, collagenase/pepsin digests were generated and analyzed as previously reported^48^. In neoplastic samples, Lys residues in the peptide containing α1 Lys-918/930 (GDKGETGEQGDRGIKGHR) were almost fully hydroxylated in a manner similar to non-neoplastic samples (Table 2). These results indicate that in canine neoplastic mammary tissues, Lys residues in the helical domain of type I collagen were over-hydroxylated compared with non-neoplastic samples at specific sites.

Next, to more specifically assess the role of Lys modification within the helical domain of type I collagen in metastatic potential, we compared the site-specific modifications in non-neoplastic, benign (adenoma) and malignant (carcinoma) mammary tumor samples. Notably, Lys hydroxylation was significantly increased in carcinoma samples compared to adenoma samples tissues (*p* < 0.05) at α1 Lys-99 (14.9% for adenoma and 22.1% for carcinoma; Table 3). Furthermore, carcinoma samples were found to have significant increases in Lys hydroxylation compared to non-neoplastic mammary tissue samples at α1 Lys-564 (12.6% for non-neoplastic and 24.0% for carcinoma). Although trends of increased Lys hydroxylation of α1 Lys-K174, α1 Lys-K219, and α2 Lys-219 in carcinoma samples compared to non-neoplastic mammary tissue samples were found, these changes did not reach statistical significance. There were no significant differences noted in site-specific modification of Lys residues when comparing benign (adenomas) and non-neoplastic mammary tissue. These results indicate that canine mammary carcinoma samples are distinguished from adenoma and non-neoplastic tissues by over-hydroxylation of specific Lys residues in the helical domain of type I collagen.

**Table 3:** Summary of site-specific modification analysis by mass spectrometry of non-cross-linked, hydroxylated and glycosylated residues in the triple helical domain of type I collagen from canine mammary tissues.

	Site occupancy (%)		
	Non-neoplastic	Adenoma	Carcinoma
**α1(I) Pro-986**			
Pro	2.30 ± 0.8	2.3 ± 1.3	3.1 ± 1.3
3-Hyp	97.7 ± 0.8	97.7 ± 1.3	96.9 ± 1.3
**α1(I) Pro-707**			
Pro	93.2 ± 0.8	90.0 ± 6.8	93.5 ± 1.5
3-Hyp	6.8 ± 0.8	10.0 ± 6.8	6.5 ± 1.5
**α2(I) Pro-707**			
Pro	89.3 ± 1.2	83.3 ± 10.8	91.2 ± 1.2
3-Hyp	10.7 ± 1.2	16.7 ± 10.8	8.8 ± 1.2
**α1(I) Lys-87**			
Lys	0.0 ± 0.0	0.0 ± 0.0	0.0 ± 0.0
Hyl	0.0 ± 0.0	0.0 ± 0.0	0.0 ± 0.0
G-Hyl	18.3 ± 1.9	22.7 ± 4.9	22.6 ± 5.9
GG-Hyl	81.7 ± 1.9	77.4 ± 4.9	77.4 ± 5.9
**α1(I) Lys-99**			
Lys	76.9 ± 2.5	81.3 ± 5.3	73.0 ± 4.8
Hyl	19.4 ± 2.2	14.9 ± 4.0	22.1 ± 4.0^†^
G-Hyl	2.7 ± 0.2	2.4 ± 0.4	3.8 ± 0.8
GG-Hyl	1.1 ± 0.3	1.4 ± 1.2	1.1 ± 0.7
**α1(I) Lys-174**			
Lys	49.3 ± 2.9	36.1 ± 12.4	33.9 ± 7.3
Hyl	44.2 ± 2.2	54.8 ± 8.7	58.3 ± 5.9
G-Hyl	5.5 ± 0.4	7.3 ± 2.9	6.6 ± 1.1
GG-Hyl	1.0 ± 0.2	1.8 ± 1.3	1.1 ± 0.7
**α1(I) Lys-219**			
Lys	84.3 ± 2.3	83.2 ± 4.8	78.0 ± 3.3
Hyl	15.7 ± 2.3	16.8 ± 4.8	22.0 ± 3.3
**α1(I) Lys-564**			
Lys	85.6 ± 1.0	76.2 ± 6.2	73.1 ± 5.5*
Hyl	12.6 ± 0.7	22.1 ± 6.0	24.0 ± 4.9*
G-Hyl	1.2 ± 0.3	1.5 ± 0.3	2.3 ± 0.6*
GG-Hyl	0.6 ± 0.1	0.2 ± 0.2	0.6 ± 0.2
**α2(I) Lys-87**			
Lys	2.5 ± 0.7	1.8 ± 0.7	1.2 ± 0.5
Hyl	0.9 ± 0.4	1.1 ± 0.1	0.7 ± 0.2
G-Hyl	11.2 ± 2.1	15.9 ± 1.0	16.3 ± 3.9
GG-Hyl	85.4 ± 2.9	81.2 ± 1.6	81.9 ± 4.1
**α2(I) Lys-174**			
Lys	13.3 ± 1.1	9.9 ± 3.6	6.6 ± 3.0
Hyl	2.2 ± 0.6	1.9 ± 1.7	1.6 ± 1.3
G-Hyl	72.5 ± 1.8	78.6 ± 4.9	82.0 ± 3.2*
GG-Hyl	11.9 ± 1.9	9.5 ± 2.9	9.7 ± 2.7
**α2(I) Lys-219**			
Lys	19.5 ± 1.3	15.1 ± 5.9	11.6 ± 1.8
Hyl	46.6 ± 2.1	60.2 ± 19.9	56.8 ± 7.1
G-Hyl	12.3 ± 0.2	10.9 ± 6.7	13.2 ± 2.3
GG-Hyl	21.6 ± 3.2	13.8 ± 8.9	18.4 ± 5.6
**α1(I) Lys-918/930**			
Lys + Lys	0.9 ± 0.1	0.5 ± 0.4	0.9 ± 0.4
Lys + Hyl	8.5 ± 0.4	6.0 ± 0.5	6.7 ± 2.0
Hyl + Hyl	90.6 ± 0.4	93.5 ± 0.9	92.4 ± 2.2

Lys hydroxylation and its glycosylation (%) represents the relative levels of Lys, Hyl, G-Hyl, and GG-Hyl (Lys + Hyl + G-Hyl + GG-Hyl = 100%), and Pro 3-hydroxylation represents the relative levels of Pro and 3-Hyp (Pro + 3-Hyp = 100%). Pro, proline; 3-Hyp, 3-hydroxyproline; Lys, lysine; Hyl, hydroxylysine; G-, galactosyl-; GG-, glucosylgalactosyl.

Values represent mean percentages ± SD (non-neoplastic, n = 3; adenoma, n = 3; carcinoma, n = 6).

*p < 0.05 between non-neoplastic and adenoma or carcinoma.

^†^*p* < 0.05 between adenoma and carcinoma via Kruskal–Wallis followed by a Dunn’s multiple comparisons test.

At seven glycosylation sites identified, α1 Lys-87, α1 Lys-99, α1 Lys-174, α1 Lys-564, α2 Lys-87, α2 Lys-174, and α2 Lys-219, the extent of glycosylation of Hyl was calculated as percentages of GG-, G-, and nonglycosylated free-Hyl in total Hyl (Supplementary Table S3). Overall, the extent of glycosylation was not significantly different among non-neoplastic and neoplastic groups (either adenoma and carcinoma separately or combined) with four exceptions. The GG-Hyl in the adenoma group was lower at α1 Lys-564 (*p* < 0.01) compared to the non-neoplastic group. In addition, G-Hyl at both α2 Lys-87 and α2 Lys-174 were increased (*p* < 0.05), while the GG-Hyl at α2 Lys-87 (*p* < 0.05) was decreased in the combined neoplastic group compared to those of non-neoplastic collagen. However, the differences are minimal, i.e. ~ 2–5%, thus, may not be biologically relevant.

### Marked Increase in Lys hydroxylation of the telo-domains of type I collagen

Next, we analyzed Lys hydroxylation in the N- and C-telo domains of type I collagen, i.e. α1(I) Lys-9^ N^, α1(I) Lys-16^C^ and α2(I) Lys-5^N^, using *Grimontia* collagenase and pepsin digestions as previously reported^48^. Hydroxylation of Lys at all three sites was significantly increased (*p* < 0.01) in mammary tumors relative to non-neoplastic mammary tissue (Table 4). The differences in this modification among the different groups are more pronounced with progression toward malignancy (non-neoplastic < adenoma < carcinoma, Table 4). At α1(I) Lys-9^ N^, only 20.1% of Lys residues was hydroxylated in non-neoplastic tissues, but it was markedly increased to 55.0% in carcinoma samples (*p* < 0.05). At α1(I) Lys-16^C^, the extent of Lys hydroxylation in carcinoma was also significantly increased (60.7% for non-neoplastic, 78.0% for adenoma, and 85.1% for carcinoma). At α2(I) Lys-5^N^, hydroxylation was 20.5% in non-neoplastic tissues while it was 43.5% and 59.2% in adenoma and carcinoma samples, respectively (Table 4). At all of these telopeptidyl sites, Lys hydroxylation in carcinoma samples was significantly higher than that of non-neoplastic samples. Although values for adenoma samples were elevated compared to non-neoplastic (and intermediate between non-neoplastic and carcinoma samples), they were not found to be significantly different from non-neoplastic tissue. These results show that by far the largest alterations in Lys hydroxylation, i.e. over-hydroxylation, occur at the telopeptidyl cross-linking sites, α1(I) Lys-9^ N^, α1(I) Lys-16^C^ and α2(I) Lys-5^N^ in mammary carcinoma samples.

**Table 4:** Lys hydroxylation in the N- and C-telopeptide domains of type I collagen from canine mammary tissues.

	Site occupancy (%)			
	Non-neoplastic	Adenoma	Carcinoma	Neoplastic
**α1(I) Lys-9**^**N**^				
Lys	79.9 ± 1.0	61.5 ± 14.7	45.0 ± 7.9*	50.5 ± 12.7^##^
Hyl	20.1 ± 1.0	38.5 ± 14.7	55.0 ± 7.9*	49.5 ± 12.7^##^
**α1(I) Lys-16**^**C**^				
Lys	39.3 ± 4.3	22.0 ± 11.1	14.9 ± 5.1*	17.3 ± 7.7^###^
Hyl	60.7 ± 4.3	78.0 ± 11.1	85.1 ± 5.1*	82.7 ± 7.7^###^
**α2(I) Lys-5**^**N**^				
Lys	79.5 ± 1.9	56.5 ± 14.6	40.8 ± 6.2*	46.0 ± 11.8^###^
Hyl	20.5 ± 1.9	43.5 ± 14.6	59.2 ± 6.2*	54.0 ± 11.8^###^

Lys hydroxylation (%) represents the relative levels of Lys and Hyl (Lys + Hyl = 100%). Lys, lysine; Hyl, hydroxylysine. ^N^, located within N-telopeptide domain; ^C^, located within C-telopeptide domain.

Values represent percentages ± S.D. (non-neoplastic, n = 3; adenoma, n = 3; carcinoma, n = 6; neoplastic, n = 9).

*p < 0.05 between non-neoplastic and malignant (carcinoma) mammary gland tumors via Kruskal–Wallis followed by a Dunn’s multiple comparisons test.

^##^*p* < 0.01; ^###^*p* < 0.001 between non-neoplastic and neoplastic (combined adenoma and carcinoma) mammary gland tumors via unpaired Student’s t-tests.

### Collagen cross-link analysis

To characterize collagen cross-links in non-neoplastic and tumor samples, we analyzed cross-links in non-neoplastic (n = 5), adenoma (*n* = 7) and carcinoma (*n* = 9) samples. In all of the samples, reducible divalent cross-links, dihydroxylysinonorleucine (DHLNL) and hydroxylysinonorleucine (HLNL), a reducible tetravalent cross-link, histidinohydroxymerodesmosine (HHMD), and nonreducible trivalent cross-links, pyridinoline (Pyr) and deoxypyridinoline (d-Pyr), were identified. The reducible cross-link DHLNL and a nonreducible cross-link, Pyr, were increased in neoplastic samples in comparison to non-neoplastic samples (Table 5a). However, for HLNL, HHMD and d-Pyr, there was no significant difference among the three groups (*p* > 0.05). Although the levels of these cross-links were markedly increased in both adenoma and carcinoma samples compared to non-neoplastic samples, these changes were found not to reach significance. The total number of aldehydes incorporated into cross-linking (DHLNL + HLNL + 2 × Pyr + 2 × d-Pyr + 2 × HHMD) was also significantly increased in neoplastic mammary tissue samples (*p* < 0.05; Table 5a), indicating that LOX is highly active in carcinoma and adenoma, producing increased amounts of cross-links. The ratio of Hyl^ald^-derived (DHLNL + Pyr + d-Pyr) to Lys^ald^-derived (HHMD) cross-links in both carcinoma and combined neoplastic samples were significantly higher compared with those of non-neoplastic samples (~ 2.7 and 2.2-fold, respectively). These results indicate that the telopeptidyl Lys in carcinoma and adenoma collagen is over-hydroxylated (Table 5b), resulting in an increase in the Hyl^ald^-derived cross-links.

**Table 5:** Levels of immature reducible cross-links (DHLNL, HLNL, and HHMD) and mature non-reducible cross-links (Pry and d-Pyr) and their ratios.

(a)						
	DHLNL	HLNL	Pyr	d-Pyr	HHMD	Total aldehydes
Non-neoplastic	0.14 ± 0.07	0.05 ± 0.01	0.26 ± 0.08	0.025 ± 0.005	0.11 ± 0.01	1.00 ± 0.15
Adenoma	0.26 ± 0.18	0.06 ± 0.02	0.41 ± 0.11	0.035 ± 0.022	0.12 ± 0.05	1.46 ± 0.43
Carcinoma	0.38 ± 0.20	0.10 ± 0.06	0.31 ± 0.21	0.037 ± 0.015	0.10 ± 0.04	1.48 ± 0.38
Neoplastic	0.30 ± 0.18^#^	0.07 ± 0.04	0.40 ± 0.13^#^	0.036 ± 0.02	0.11 ± 0.05	1.46 ± 0.41^#^

(b)						
				(DHLNL + Pyr + d-Pyr)/HHMD		
Non-neoplastic				3.87 ± 0.98		
Adenoma				6.43 ± 1.96		
Carcinoma				10.04 ± 5.4*		
Neoplastic				8.46 ± 4.53^#^		

(a, b) DHLNL, dihydroxylysinonorleucine; HLNL, hydroxylysinonorleucine; HHMD, histidinohydroxymerodesmosine; Pyr, pyridinoline; d-Pyr, deoxypyridinoline. Total aldehydes = DHLNL + HLNL + 2 × Pyr + 2 × d-Pyr + 2 × HHMD. Values represent moles/mole collagen ± S.D (non-neoplastic, n = 5; adenoma, n = 7; carcinoma, n = 9; neoplastic, n = 16).

*p < 0.05 between non-neoplastic and malignant (carcinoma) mammary gland tissues via Kruskal–Wallis followed by a Dunn’s multiple comparisons test.

^#^*p* < 0.05 between non-neoplastic and neoplastic (combined adenoma and carcinoma) mammary gland tissues via unpaired Student’s *t*-tests.

### Gene expression analysis

**Figure 2:**
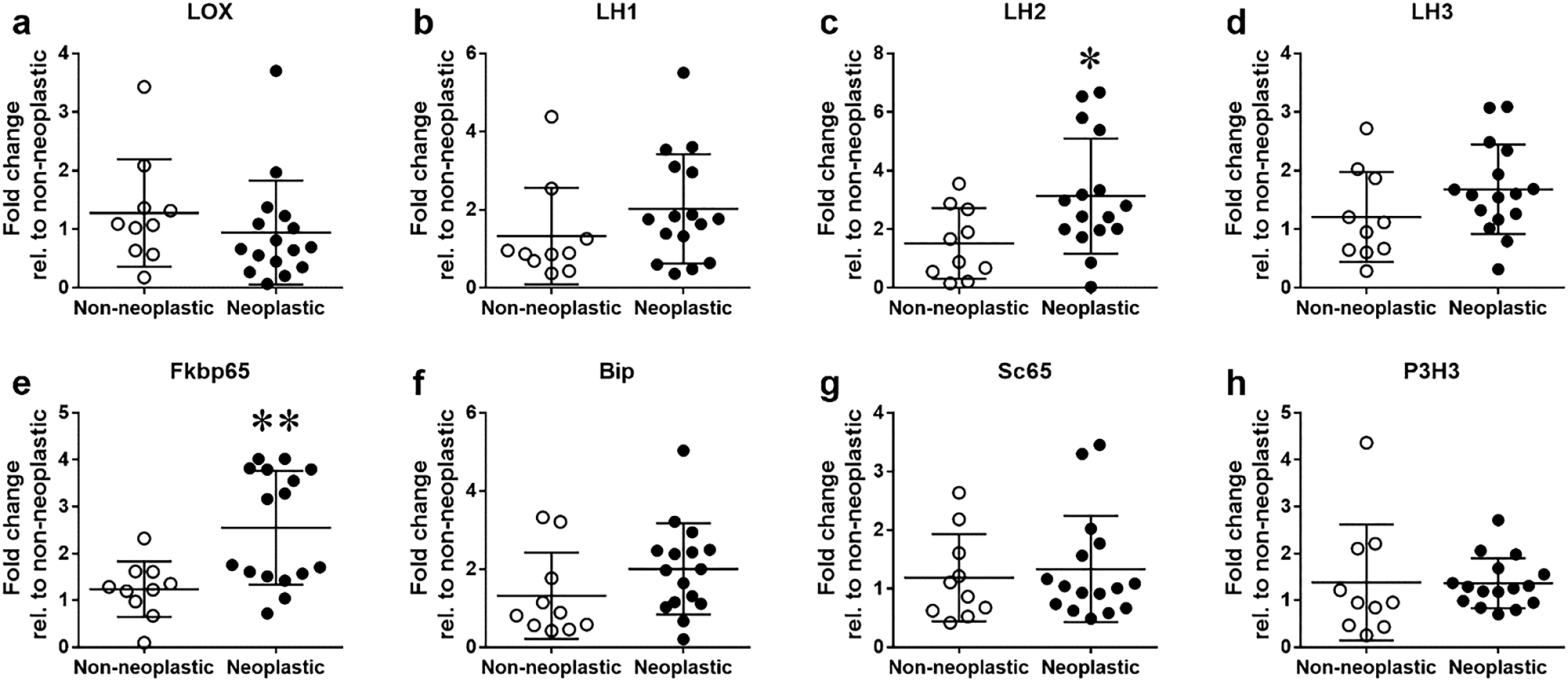
Expression levels of cross-linking enzymes, chaperones. and modulators. (**a**–**h**) mRNA expression in canine mammary tumor tissue samples was analyzed using quantitative real-time PCR for genes encoding LOX (**a**), LH1-3 (**b**–**d**), Fkbp65, (**e**), Bip (**f**), Sc65 (**g**) and P3H3 (**h**). Gene expression was compared between non-neoplastic (n = 10) and neoplastic samples (n = 16). *p < 0.05; **p < 0.01 via unpaired Student’s *t*-tests. Data are presented as means ± SD.

Given the quantitative and qualitative changes in collagen cross-links in neoplastic samples (Table 5), we evaluated the mRNA expression levels of key enzymes for cross-linking, i.e. LOX and LH1-3 (encoded by *Plod1–3*), by quantitative real-time PCR analysis. In addition, the recently reported LH-associated chaperones were also examined. The chaperones included: 1. LH2 modulators: FK506-binding protein 65 (Fkbp65; encoded by *Fkbp10*) (a positive modulator for LH2) and immunoglubulin heavy-chain-binding protein (Bip), and 2. LH1 modulators: Synaptonemal Complex 65 (Sc65) and prolyl 3-hydroxylase 3 (P3H3). Gene expression was compared between non-neoplastic and neoplastic samples (Fig. 2). While most genes examined showed trends for increased expression in neoplastic compared to non-neoplastic tissue, LH2 and its positive modulator Fkbp65, both specifically involved in telopeptidyl Lys hydroxylation, had significantly increased expression in neoplastic vs non-neoplastic tissue (*p* < 0.05 and *p* < 0.01, respectively).

## Discussion

### Validation of a model for human breast cancer: biological significance of collagen modifications in canine mammary neoplasia

In this study, we demonstrated key changes in fibrillar collagen consistent with a tumor-permissive microenvironment in canine mammary gland neoplasia. Building upon our previous work which defined intratumoral collagen fiber width as a predictive biomarker in dogs with mammary gland carcinoma, we show that mammary gland carcinoma biopsies contain a greater distribution of fibers of increased width compared to benign adenomas and non-neoplastic mammary tissue. Beyond this noted structural change in collagen fibers, we further demonstrate qualitative and quantitative alterations of collagen post-translational modifications in neoplastic mammary tissues. Notably, Lys residues in collagen are over-hydroxylated compared to non-neoplastic samples. However, the extent of this over-hydroxylation is far more pronounced in the both N- and C-telo domains of the type I collagen molecule. The extent of glycosylation, both mono- and di-glycosylation, are mostly unchanged among the groups. Consistent with these observations, collagen cross-links in neoplastic samples are enriched in the Hyl^ald^-derived, stable cross-links. The results of gene expression analyses also demonstrated that *Plod2*, encoding LH2, and *Fkbp10*, encoding the LH2 chaperone FKBP65, are both significantly increased in neoplastic tissues compared to those of non-neoplastic tissues. In neoplastic samples, not only were the type of cross-links altered but also the number of total aldehydes involved in cross-links increased, which is consistent with our recent report on oral squamous cell carcinoma^18^. These alterations most likely create stiffened ECM in the tumor microenvironment^1,34,59^.

Fibrillar type I collagen is a major structural component in the tumor microenvironment, which regulates cancer progression^60–62^ by modulating cancer cell differentiation, proliferation, migration, survival, and metastasis^60–65^. In fact, increased mammographic density, which correlates with an increase in stromal collagen content and alignment, remains one of the strongest predictors of breast cancer risk in women^66^. More recent studies have focused on specific tumor-associated collagen signatures (TACS) and fiber characteristics which are capable of predicting disease progression in murine models of breast cancer and human and veterinary patients^13–15,67,68^. An expanding body of literature has further begun to define a critical role for collagen cross-links in regulating such collagen phenotypes. It has been well documented that LOX family members, especially LOX and LOXL2, are elevated and associated with tumor progression^28,29,69,70^. Elevation of these enzymes, which catalyze the formation of Lys^ald^ and Hyl^ald^ that initiate cross-linking, would increase the amounts of cross-links, thus, contributing to the overall stiffening of tumor stroma to facilitate tumor cell invasion^71^. Recently, LH2, another collagen modifying enzyme that determines the type/stability of cross-links^23^, has been implicated in cancer metastasis and decreased survival rate in various cancer models, including breast cancer^18,31–34,72–74^. Among these reports, only a few studies^18,34^ performed collagen cross-link analysis in tumors to evaluate the outcome of the elevated LH2 gene expression. To the best of our knowledge, no study has shown how Lys residues at the specific loci in type I collagen are modified as a result of abnormal modifying enzyme levels, and how ER chaperones that modulate LH activities are affected in tumors.

In order to elucidate the molecular mechanisms driving tumorigenesis and metastatic potential as well as to develop therapeutic strategies for human breast cancer, a number of animal models such as mice^75^, rats^76,77^, cats^16,76–80^, and dogs^9,81–84^ have been developed. Given the protracted timeline and staggering expense of cancer therapies (estimated US ~ $1.8 billion to bring a new therapeutic from target validation to market)^85^, compounded by the high rate of failure (59% of anti-neoplastic drugs entering Phase III clinical trials)^86,87^, it is imperative to examine toxicity and efficacy in preclinical trials using a model that most closely mimics breast cancer in women. As detailed above, the canine model has been recognized to have superior fidelity to the human disease when compared to other laboratory models of breast cancer^11,36–43,81,88^. Furthermore, client-owned dogs are a genetically diverse population, are of large body size, share similar carcinogenic environmental risk factors, and undergo similar oncologic diagnostics and therapeutics to their human counterparts^83,89^. Heterogeneity at both the patient and tumor levels more accurately models that in corresponding human patients than laboratory research animals. Until recently, investigations of cancer development and progression have focused on mechanisms within neoplastic cells that drive uncontrolled growth and metastasis. In a recent paradigm shift, we now realize the growth, mobility and spread of cancer depends on the support of non-malignant cells and the ECM, particularly fibrillar collagens, in the surrounding tumor stroma collectively referred to as the tumor microenvironment. Notably, similar to that seen in human breast cancer, canine mammary carcinomas elicit a robust desmoplastic response which is often lacking in murine models^90^. Recent studies have begun to further characterize the cancer-associated stroma, particularly cancer associated fibroblasts and ECM components such as fibrillar collagens, in canine mammary gland tumors^12,42,91–93^, which is a critical step for validating the stromal response in this model for extrapolation to human breast cancer. Although LOX has been previously reported to be overexpressed in mammary carcinomas compared to normal mammary tissue, there has yet to be collagen cross-linking analysis performed in canine mammary tumors nor has the role of LHs been examined^94^. Given the recent finding that LH2 expression in breast cancer biopsies from women significantly correlates with disease specific mortality^32^, defining its potential role in malignancy in canine mammary tumors is critical to further characterize this important model, as well as to advance our understanding of the role of specific features of the stromal microenvironment on breast cancer.

### Key features of collagen microarchitecture in non-neoplastic and neoplastic canine mammary tissue

Previous work from our laboratory identified an inverse correlation between collagen density, as well as fiber width, length and straightness, with canine mammary carcinoma patient overall survival time^12^. Here, we show significant increases in collagen fiber width and length in carcinoma samples compared to non-neoplastic or adenoma samples. Although we did not see a significant increase in collagen density or fiber straightness in carcinoma samples relative to benign and non-neoplastic samples, we believe the inclusion of a large number of low-grade tumors from long-lived patients may have skewed the carcinoma sets to appear more benign than if it had contained a higher population of biologically aggressive tumors. Even with a diverse and small cohort of carcinoma samples, it is not surprising that fiber width is different between these and non-malignant samples as we previously showed that collagen fiber width can predict poor outcome in canine mammary tumor patients, even when used in multivariate analysis taking clinical parameters into account. Increased collagen fiber width in the peritumor stroma in human colonic cancer^95^ and in the stroma immediately surrounding neoplastic epithelium of pancreatic ductal adenocarcinoma samples was also recognized to favor cancer progression^96^ and increased intratumoral collagen width was a powerful predictor of poor outcome in human gastric cancer^97^. The increased fiber width has been shown to correlate with change in cancer cell shape, formation of invadopodia and increased migration^98,99^ and has recently been shown to mechanically activate myofibroblasts^100^. Although myofibroblast density was not examined here, an increased presence of myofibroblasts has been previously shown to correlate with malignant progression in canine mammary tumors^42^.

### Increased LH2 and FKBP65 expression in canine mammary neoplasia is consistent with over-hydroxylation of Lys in type I collagen telopeptides and cross-linking profile

Next, we investigated the molecular phenotype of collagen by quantifying cross-links and site-specific Lys modifications in type I collagen in both neoplastic (adenoma and carcinoma) and non-neoplastic canine mammary tissues. By determining the relative abundance of Lys modifications at the specific sites of type I collagen, we found that hydrolation at all Lys residues in the N- and C-telo domains was markedly elevated in neoplastic compared to non-neoplastic samples. To the best of our knowledge, this is the first demonstration in cancer samples of any species that Lys hydroxylation at these cross-linking sites is highly elevated. This is consistent with increased *Plod2* and *Fkbp10* gene expression (Fig. 2) and cross-link data (Table 5). Obviously, further studies are warranted to confirm these data by increasing the sample size with various clinical outcomes to determine whether Lys modification confers prognostic information in canine mammary gland tumors. It is now clear that LH activity is regulated by a number of ER-chaperone complexes. LH1 activity, for instance, is regulated by a complex composed of Sc65, P3H3 and CypB (cyclophilin B). The formation of this complex facilitates LH1 activity at the helical cross-linking sites^101,102^. For LH2, it has been reported that ER components Bip, Hsp47 (heat shock protein 47), Fkbp65 and LH2 form a complex to regulate LH2 activity at the C-telopeptidyl cross-linking site^103^. Of these, FKBP65 functions as a positive modulator for LH2 by specifically binding LH2 and facilitating its dimerization that is crucial for its enzymatic activity^104^. On the contrary, HSP47 may function as a negative regulator either by impeding LH2 access to the telopeptidyl Lys or by blocking LH2 dimerization by FKBP65^103^. Bip also may play a role in the formation of this chaperone complex and the absence of Bip leads to over-hydroxylation of the telopeptidyl Lys residues^103^. Thus, our findings of increased *Plod2* and *Fkbp10* gene expression levels seen in neoplastic samples favor the LH2-catalized telopeptidyl Lys hydroxylation leading to a clear increase of the ratio of Hyl^ald^- to Lys^ald^-derived cross-links (Table 5b). To determine the ratio, we used HHMD as the Lys^ald^-derived cross-link. Though the natural occurrence of this tetravalent reducible cross-link has been controversial since it was isolated by Tanzer’s group 46 years ago until now^105^, two things are certain: (1) HHMD is an unstable, reducible cross-link and (2) it contains an aldol formed by two Lys^ald^ residues. Thus, LH2-catalyzed Lys hydroxylation is absent to form this cross-link. In adenoma samples, Lys hydroxylation in all telopeptides also showed an increasing trend though did not reach significant levels (Table 3). Further studies on the role of this complex interplay among ER-chaperones including HSP47 and CypB that modulate LH2 activity^51^ in cancer biology are warranted. As stated above, LH3 level and its activity may not be altered significantly in carcinoma.

In conclusion, our study revealed that, in canine mammary neoplasia, due to the highly expressed specific Lys modifying enzymes and associated LH-chaperone components, all teloepeptidyl Lys in type I collagen are significantly overhydroxylated, resulting in the increases of LH2-mediated stable collagen cross-links. Thus, although the interpretation of this study is limited by the relatively small sample size, these results underscore the critical role of collagen post-translational modifications in canine mammary gland tumors and provide premise for both future in-depth studies examining their ability to direct clinical behavior of mammary tumors and the use of this model in stromal targeting translational studies in a One Health approach to breast cancer. Further understanding how LHs are activated during cancer development and metastasis may aid in the development of novel LH inhibitors as a therapeutic strategy to improve clinical outcomes in both veterinary and human breast cancer patients.

## Supplementary Information

Supplementary Information

## Data Availability

The datasets generated during and/or analyzed during the current study are available from the corresponding authors upon request.
