# Supplementary material for: Collagen molecular phenotypic switch between non-neoplastic and neoplastic canine mammary tissues
Source: Sci Rep. 2021 Apr 21;11:8659. doi: 10.1038/s41598-021-87380-y (PMC8060395; doi:10.1038/s41598-021-87380-y)
Supplement: Supplementary file 1 — Supplementary Information [file 41598_2021_87380_MOESM1_ESM.docx]

**Supplementary Information**

**Collagen molecular phenotypic switch between non-neoplastic and neoplastic canine mammary tissues**

Masahiko Terajima^1*^, Yuki Taga^2*^, Becky K. Brisson^3*^, Amy C. Durham^4^, Kotaro Sato^5^, Katsuhiro Uzawa^6^, Tomoaki Saito^6^, Shunji Hattori^2^, Karin U. Sørenmo^7^, Mitsuo Yamauchi^1#^, Susan W. Volk^3,7#^

^1^Division of Oral and Craniofacial Health Sciences, Adams School of Dentistry, University of North Carolina at Chapel Hill, United States of America

^2^Nippi Research Institute of Biomatrix, Japan

^3^Department of Clinical Sciences and Advanced Medicine, School of Veterinary Medicine, University of Pennsylvania, United States of America

^4^Department of Pathobiology, School of Veterinary Medicine, University of Pennsylvania, United States of America

^5^Department of Oral and Maxillofacial Surgery, Nagoya University Graduate School of Medicine, Japan

^6^Department of Oral Science, Graduate School of Medicine, Chiba University, Japan

^7^Department of Biomedical Sciences, School of Veterinary Medicine, University of Pennsylvania, United States of America

^*^ These authors contributed equally to this work.

^#^Co-corresponding authors: Mitsuo Yamauchi and Susan W. Volk

Email: [Mitsuo_Yamauchi@unc.edu](mailto:Mitsuo_Yamauchi@unc.edu) and [swvolk@vet.upenn.edu](mailto:swvolk@vet.upenn.edu)

**Supplementary Table 1.** Patient Sample Information.

| **Patient** | **Breed** | **Neoplastic** | **Tumor Category** | **Diagnosis** | **Grade** | **OS** | **DFS** | **Analysis** | | | | |
| --- | --- | --- | --- | --- | --- | --- | --- | --- | --- | --- | --- | --- |
|  |  |  |  |  |  |  |  | **SHG** | **Amino Acid** | **Mass Spec** | **Cross-link** | **qPCR** |
| 1 | BULL | N | non-neoplastic | lobular hyperplasia |  |  |  | x | x | x | x | x |
| 2 | MIXB | N | non-neoplastic | lobular hyperplasia |  |  |  | x | x | x | x | x |
| 3 | BEAG | N | non-neoplastic | lobular hyperplasia |  |  |  | x | x | x | x | x |
| 4 | APBT | N | non-neoplastic | lobular hyperplasia |  |  |  | x | x |  | x | x |
| 5 | APBT* | N | non-neoplastic | normal mammary gland |  |  |  |  | x |  | x | x |
| 6 | HUSK | N | non-neoplastic | lobular hyperplasia |  |  |  |  |  |  |  | x |
| 7 | AKIA | N | non-neoplastic | lobular hyperplasia |  |  |  |  |  |  |  | x |
| 8 | MIXB | N | non-neoplastic | lobular hyperplasia |  |  |  |  |  |  |  | x |
| 9 | MIXB | N | non-neoplastic | lobular hyperplasia |  |  |  |  |  |  |  | x |
| 10 | AKIA | N | non-neoplastic | lobular hyperplasia |  |  |  |  |  |  |  | x |
| 11 | MIXB | N | adenoma | complex adenoma |  |  |  | x | x | x | x | x |
| 12 | MIXB | N | adenoma | complex adenoma |  |  |  | x | x | x | x | x |
| 13 | MIXB^#^ | N | adenoma | adenoma |  |  |  | x | x |  | x | x |
| 14 | MIXB | N | adenoma | complex adenoma |  |  |  | x | x |  | x | x |
| 15 | MIXB | N | adenoma | complex adenoma |  |  |  |  | x |  | x | x |
| 16 | MIXB | N | adenoma | adenoma |  |  |  |  | x |  | x | x |
| 17 | CHIU | N | adenoma | cystadenoma |  |  |  |  | x | x | x |  |
| 18 | MINP | N | adenoma | complex adenoma |  |  |  |  |  |  |  | x |
| 19 | MALT | N | adenoma | complex adenoma |  |  |  |  |  |  |  | x |
| 20 | PEKE | N | adenoma | complex adenoma |  |  |  |  |  |  |  | x |
| 21 | MIXB | N | adenoma | complex adenoma |  |  |  |  |  |  |  | x |
| 22 | CHIU | Y | carcinoma | carcinoma arising in adenoma | I |  |  |  | x | x | x | x |
| 23 | DACH | Y | carcinoma | carcinoma | I | 908 | 908 | x | x | x | x | x |
| 24 | MIXB^#^ | Y | carcinoma | carcinoma | III | 382 | 121 | x | x | x | x | x |
| 25 | YRKT | Y | carcinoma | carcinoma arising in adenoma | I | 1118 | 544 | x | x | x | x | x |
| 26 | GSPD | Y | carcinoma | carcinoma arising in adenoma | I | 433 | 433 | x | x |  | x | x |
| 27 | DACH | Y | carcinoma | carcinoma | I | 167 | 113 | x | x | x | x |  |
| 28 | APBT* | Y | carcinoma | carcinoma | II | 711 | 706 | x | x | x | x |  |
| 29 | BOST | Y | carcinoma | carcinoma | II | 1145 | 614 | x | x |  | x |  |
| 30 | ROTW | Y | carcinoma | carcinoma | II | 819 | 819 | x | x |  | x |  |
| 31 | MALT | Y | carcinoma | carcinoma | II | 118 | 118 | x |  |  |  |  |
| 32 | MIXB | Y | carcinoma | anaplastic carcinoma | III | 109 | 109 | x |  |  |  |  |
| 33 | DACH | Y | carcinoma | carcinoma | II | 595 | 595 |  |  |  |  | x |

English Bulldog (BULL), Beagle (BEAG), American Pit Bull Terrier (APBT), mixed breed dog (MIXB), German Shepherd (GSPD), Dachshund (DACH), Rottweiler (ROTW), Boston Terrier (BOST), Chihuahua (CHIU), Yorkshire Terrier (YRKT), Huskey (HUSK), Akita (AKIA), Maltese (MALT), Pekingese (PEKE). Yes (Y); No (N). Tumor Grade, overall survival time (OS) and disease-free survival time (DFS) are given for carcinoma samples. Multiple tissue samples were obtained from dogs (N=2) denoted with * or ^#^. OS and DFS are not reported for patient #22, as this patient was euthanized for co-morbidities prior to surgery (tumor biopsy performed at necropsy).

**Supplementary Table 2.** Primer sequences

| Protein | Primer | Sequence |
| --- | --- | --- |
| LOX | cLOXF | CGG ATA CGG CAC CGG CTA TT |
|  | cLOXR | CTC TGC AGC GCA TCT CAG GT |
| LH1 | cPLOD1F | GGG CGA CTT ACG CAC TAC CA |
|  | cPLOD1R | CGC AGA GGT CCA AGG TAG CC |
| LH2 | cPLOD2F | GGA CAC AGG ATA ATG GCT GCA C |
|  | cPLOD2R | CCG AGG TAG GAA AGG GGT TGG |
| LH3 | cPLOD3F | CAT GAG CCC CAC ATC GCA GA |
|  | cPLOD3R | CTG GGT CCT GCC GAC AAC TG |
| FKBP65 | cFkbp10F | TTC GTG TGG CAT GAG GAC CC |
|  | cFkbp10R | GTC CTT TGC CCT CGC TGA CT |
| Bip | cBipF | TCA TCG CCA ACG ATC AGG GC |
|  | cBipR | TCG GGG TTG GAA GTG AGC TG |
| Sc65 | cSc65F | CTA CGA GGC CGT GTT CCT CAG |
|  | cSc65R | CAG ACA TCG GGC AAA GAT GGC |
| P3H3 | cP3h3F | ACA CTT GCA GAT GCG GGA GG |
|  | cP3h3R | GCC CTC ATC ATA GGC TGC CC |

c, canine. All sequence 5’-3’

**Supplementary Table 3.** Glycosylation of hydroxylysine residues estimated by mass spectrometry of non-cross-linked glycosylated residues in canine mammary tissues.

|  |  | | **Site occupancy (%)** | | | |  | | |  |  |  |
| --- | --- | --- | --- | --- | --- | --- | --- | --- | --- | --- | --- | --- |
|  |  | | **Non-neoplastic** | **Adenoma** | **Carcinoma** | | | **Neoplastic** | | |  |  |
| α1(I) Lys-87 | Hyl | | 0.0 ± 0.0 | 0.0 ± 0.0 | | 0.0 ± 0.0 | | | 0.0 ± 0.0 | | |  |
|  | G-Hyl | | 18.3 ± 1.9 | 22.7 ± 4.9 | | 22.6 ± 5.9 | | | 22.6 ± 5.2 | | |  |
|  | GG-Hyl | | 81.7 ± 1.9 | 77.4 ± 4.9 | | 77.4 ± 5.9 | | | 77.4 ± 5.2 | | |  |
| α1(I) Lys-99 | Hyl | | 83.7 ± 0.5 | 79.8 ± 2.9 | | 81.9 ± 2.6 | | | 81.2 ± 2.7 | | |  |
|  | G-Hyl | | 11.7 ± 1.5 | 13.5 ± 3.8 | | 14.0 ± 0.8 | | | 13.9 ± 2.6 | | |  |
|  | GG-Hyl | | 4.5 ± 1.0 | 6.6 ± 4.6 | | 4.1 ± 2.4 | | | 4.9 ± 3.2 | | |  |
| α1(I) Lys-174 | Hyl | | 87.2 ± 0.6 | 86.2 ± 3.6 | | 88.3 ± 1.5 | | | 87.6 ± 2.4 | | |  |
|  | G-Hyl | | 10.8 ± 0.3 | 11.0 ± 2.5 | | 10.0 ± 0.6 | | | 10.3 ± 1.4 | | |  |
|  | GG-Hyl | | 2.0 ± 0.4 | 2.7 ± 1.8 | | 1.7 ± 0.9 | | | 2.0 ± 1.2 | | |  |
| α1(I) Lys-564 | Hyl | | 87.3 ± 1.8 | 92.6 ± 1.0* | | 89.2 ± 1.8 | | | 90.3 ± 2.3 | | |  |
|  | G-Hyl | | 8.6 ± 1.5 | 6.6 ± 1.5 | | 8.5 ± 1.2 | | | 7.9 ± 1.6 | | |  |
|  | GG-Hyl | | 4.1 ± 0.4 | 0.9 ± 0.8** | | 2.3 ± 0.7 | | | 1.8 ± 1.0^##^ | | |  |
| α2(I) Lys-87 | Hyl | | 1.0 ± 0.4 | 1.1 ± 0.1 | | 0.7 ± 0.2 | | | 0.8 ± 0.2 | | |  |
|  | G-Hyl | | 11.5 ± 2.2 | 16.2 ± 1.2 | | 16.5 ± 4.0 | | | 16.4 ± 3.2^#^ | | |  |
|  | GG-Hyl | | 87.5 ± 2.4 | 82.7 ± 1.1 | | 82.8 ± 4.1 | | | 82.7 ± 3.3^#^ | | |  |
| α2(I) Lys-174 | Hyl | | 2.6 ± 0.7 | 2.1 ± 1.8 | | 1.8 ± 1.4 | | | 1.9 ± 1.5 | | |  |
|  | G-Hyl | | 83.7 ± 2.5 | 87.2 ± 1.9 | | 87.8 ± 2.2 | | | 87.6 ± 2.0^#^ | | |  |
|  | GG-Hyl | | 13.7 ± 2.1 | 10.7 ± 3.6 | | 10.4 ± 2.8 | | | 10.5 ± 2.9 | | |  |
| α2(I) Lys-219 | Hyl | | 57.9 ± 3.4 | 70.2 ± 18.9 | | 64.3 ± 8.3 | | | 66.3 ± 11.9 | | |  |
|  | G-Hyl | | 15.3 ± 0.5 | 13.1 ± 8.2 | | 14.9 ± 2.6 | | | 14.3 ± 4.6 | | |  |
|  | GG-Hyl | | 26.8 ± 3.6 | 16.7 ± 10.9 | | 20.8 ± 6.2 | | | 19.4 ± 7.6 | | |  |
|  | |  | | | | | | | | | | |

Glycosylation of Hyl residues (%) represents the relative levels of Hyl, G-Hyl, and GG-Hyl (Hyl + G-Hyl + GG-Hyl = 100%). Hyl, hydroxylysine; G-, galactosyl-; GG-, glucosylgalactosyl.

Values represent percentages ± S.D. (non-neoplastic, n=3; adenoma, n=3; carcinoma, n=6; neoplastic, n=9).

*p<0.05; **p<0.01 between non-neoplastic and adenoma via Kruskal-Wallis followed by a Dunn’s multiple comparisons test. ^#^p<0.05; ^##^p<0.01 between non-neoplastic and neoplastic (combined adenoma and carcinoma) mammary gland tissues via unpaired student t-tests.
